# Evaluation of the pressure ulcers risk scales with critically ill patients: a prospective cohort study^1^

**DOI:** 10.1590/0104-1169.0144.2521

**Published:** 2015

**Authors:** Andressa Tomazini Borghardt, Thiago Nascimento do Prado, Thiago Moura de Araújo, Noemi Marisa Brunet Rogenski, Maria Edla de Oliveira Bringuente

**Affiliations:** 2 MSc, RN, Hospital Universitário Cassiano Antônio Moraes, Universidade Federal do Espirito Santo, Vitória, ES, Brazil; 3 Doctoral student, Universidade Federal do Espirito Santo, Vitória, ES, Brazil. Assistant Professor, Departamento de Enfermagem, Universidade Federal do Espirito Santo, Vitória, ES, Brazil; 4 PhD, Adjunct Professor, Instituto de Ciências da Saúde, Universidade da Integração Internacional da Lusofonia Afro-Brasileira, Redenção, CE, Brazil; 5 PhD, RN, Hospital Universitário, Universidade de São Paulo, São Paulo, SP, Brazil; 6 PhD, Associate Professor, Universidade Federal do Espirito Santo, Vitória, ES, Brazil

**Keywords:** Pressure Ulcer, Risk Assessment, Scales, Nursing Care

## Abstract

**AIMS:**

to evaluate the accuracy of the Braden and Waterlow risk assessment scales in critically ill inpatients.

**METHOD:**

this prospective cohort study, with 55 patients in intensive care units, was performed through evaluation of sociodemographic and clinical variables, through the application of the scales (Braden and Waterlow) upon admission and every 48 hours; and through the evaluation and classification of the ulcers into categories.

**RESULTS:**

the pressure ulcer incidence was 30.9%, with the Braden and Waterlow scales presenting high sensitivity (41% and 71%) and low specificity (21% and 47%) respectively in the three evaluations. The cut off scores found in the first, second and third evaluations were 12, 12 and 11 in the Braden scale, and 16, 15 and 14 in the Waterlow scale.

**CONCLUSION:**

the Braden scale was shown to be a good screening instrument, and the Waterlow scale proved to have better predictive power.

## Introduction

The occurrence of pressure ulcers (PUs) is still a common phenomenon in many healthcare settings, constituting an injury that mainly affects critically ill patients^(^ 1 ^)^ and contributes to the increased risk of hospital complications^(^ 2 ^-^ 3 ^)^. Despite the technological and scientific advances and the improvement of services and healthcare, the incidence of pressure ulcers has remained high and varies widely, from 23.1% to 59.5%, mainly in Brazilian studies and among intensive care unit patients^(^ 4 ^-^ 5 ^)^.

The pressure ulcer is defined as a lesion of the skin or underlying tissue, usually over a bony prominence, as a result of pressure associated with friction forces. Ulcers are classified into six categories: category I is characterized by non-blanchable erythematous lesions in intact skin over areas of bony prominence; category II is characterized by partial loss of the cutaneous surface, presenting as an abrasion, blister or with shallow deepithelization; category III is characterized by total skin loss, involving the subcutaneous tissue area; category IV is characterized by extensive tissue loss and exposure of the muscle, bone and/or underlying tendons; the Unclassified category is characterized by complete loss of tissue, being filled with necrotic tissue or eschar, and finally, the Suspected Deep Tissue Injury (SDTI) category includes ulcers that present dark red or purple areas in the intact skin or phlyctena with blood^(^ 6 ^)^. 

The development of pressure ulcers is often rapid and causes complications for the hospitalized individual, as well as prolonging the treatment and rehabilitation, this diminishes the quality of life, causes pain and increases mortality^(^ 7 ^)^. Given the severity of the problem for the patient, the family and the institution, the need to prevent PUs is undeniable^(^ 8 ^)^.

The presence of PUs is still negatively associated with the quality of nursing care^(^ 3 ^,^ 6 ^)^, however, this is a multifactorial problem, which includes extrinsic factors related to the physical exposure of the patient, and intrinsic factors inherent to the clinical condition, such as hemodynamic changes, anemia, malnutrition, and smoking, among others^(^ 3 ^,^ 8 ^-^ 9 ^)^. Careful and periodic evaluation of the patient at risk for PU development is essential in nursing practice. Therefore, various risk assessment instruments have been developed and some of them have been validated in Brazil, with the Braden and Waterlow scales among the most commonly used^(^ 10 ^)^.

Risk assessment scales establish, through the score, the probability of the occurrence of PU in a patient, based on a series of parameters considered risk factors^(^ 11 ^)^. These scales include the general condition and evaluation of the skin, mobility, moisture, incontinence, nutrition, and pain, among other factors^(^ 6 ^)^.

The Waterlow scale has evaluative aspects of great relevance for the study of hospitalized patients. This scale assesses seven main topics: weight/height (BMI), visual evaluation of skin in risk areas, gender/age, continence, mobility, appetite and medications, as well as four items that constitute special risk factors: tissue malnutrition, neurological deficit, length of surgery over two hours, and trauma to the lumbar region. The higher the score, the higher the risk of developing pressure ulcers, with patients also stratified into risk groups according to the score^(^ 10 ^)^.

Regarding the Braden scale, this is based on the pathophysiology of the pressure ulcers and allows the evaluation of important aspects for the formation of ulcers, according to six parameters: sensory perception, moisture, mobility and activity, nutrition, friction, and shear. The first five sub-scales have a score ranging from 1 to 4, while the scores of the friction and shear sub-scales range from 1 to 3. The sum of scores of each sub-scale ultimately allows stratification into groups, with lower values indicating worse conditions^(^ 12 ^)^. 

The scales are useful, they complement each other and they provide benefits in the systematic evaluation of the patient. In critically ill patients the use of these instruments should occur daily as a result of changes in the clinical conditions requiring the implementation of appropriate preventive behaviors after the diagnosis of risk^(^ 13 ^)^. The role of the nurse in assessing the risk supports integral and individualized care for the patient and family^(^ 14 ^)^ and provides essential information for the care plan, ensuring effective multidisciplinary communication^(^ 6 ^)^.

In order to describe the applicability of the risk assessment scales in different populations, the aim of this study was to evaluate the accuracy of the Braden and Waterlow risk assessment scales with critically ill inpatients. 

## Methods

This prospective cohort study was performed with 55 inpatients, admitted between March and June 2013, in intensive care units (Intermediate Surgical Intensive Care Unit and Intensive Care Center) of the Cassiano Antonio Moraes University Hospital (HUCAM), which treats surgical and general medical patients. The following inclusion criteria were used: to be 18 years of age or more; to have no pressure ulcer on admission, and the exclusion criteria were, to not have undergone laboratory examinations and to have had less than three consecutive evaluations.

After approval by the Research Ethics Committee of the institution (CAAE No. 07402912. 2.0000. 5071), data were collected daily by the researcher. Document analysis techniques were used, as well as an interview with the patient and family member/guardian (when the patient was sedated or had cognitive impairment) and evaluation of the skin and ulcers when present. The instrument used was a composite form of four parts: the first collects sociodemographic data; the second covers general clinical data, metabolic data and factors related to the injury; the third constitutes the clinical evaluation of risk for PU development through the Waterlow and Braden scales (upon admission and every 48 hours), and the fourth part contains data for the evaluation and classification of the ulcers according to the prevention and treatment guidelines of the National Pressure Ulcer Advisory Panel - NPUAP^(^ 6 ^)^. When the presence of pressure ulcers was verified, the nurse of the sector was informed so that the therapeutic necessary procedures for the patient could be implemented. Patient evaluation and the application of the scales were performed daily until discharge or death, however, for the purpose of analysis the first three evaluations were used.

The variables analyzed related to sociodemographic data were: gender (male and female); age (more or less than 60 years); skin color (white or non-white); hospital sector (Intensive Care Unit ─ ICU or Intermediate Unit ─ IU); marital status (married, single, widowed or divorced); schooling (illiterate, elementary, high school or higher education) and work status (active or retired). The general clinical data were: length of hospitalization (less than 10 days or more than/equal to 10 days); type of hospitalization (clinical or surgical); clinical diagnosis (gastrointestinal, cardiorespiratory, urodynamic, rheumatological/hematological or neuroinfectious); presence or absence of diabetes mellitus, smoking or congestive heart failure (CHF); use or not of mechanical ventilation, norepinephrine, or sedation. Factors related to the ulcer, i.e., the categories (I, II, III, IV, SDTI and Unclassified), number of ulcers, and locations (sacral, trochanteric, calcaneal, malleolus, occipital and elbow) were described.

In the use of the scales, the risk was assigned according to the stratification determined by the scale. In the Waterlow scale patients can be stratified into three groups, according to the score: at risk (10 to 14 points), high risk (15 to 19 points) and very high risk of ulcer development (≥20 points)^(^ 10 ^)^ and in the Braden scale the total score corresponds to the groups: > 16 points, no risk; 12 to 15 points, moderate risk; <11 points, high risk^(^ 12 ^)^. 

The analysis process of the study data was divided into two stages. In the first stage the PU incidence calculation was performed; in the second the evaluation was performed and the accuracy of the Braden and Waterlow scales was calculated using the statistical package STATA Version 11. 0 (Stata Corp, College Station, TX, USA, 2001). Seeking to standardize the evaluations, the scores obtained from the application of the scales in the first three evaluations were used, i.e., 24, 48 and 72 hours after admission. These evaluations shortly after the hospitalization of the patients in intensive care are critical, as, in many cases, there is an indication of restriction to the bed, use of vasoactive drugs, mechanical ventilation and sedation.

The variables were presented as absolute frequencies and percentages, as well as measures of central tendency. The evaluation of the accuracy of the scales was performed through the calculations of the diagnostic test properties, sensitivity, specificity, positive predictive value, negative predictive value, the likelihood ratio for a positive test and likelihood ratio for a negative test. 

## Results

**Figure 1 -:**
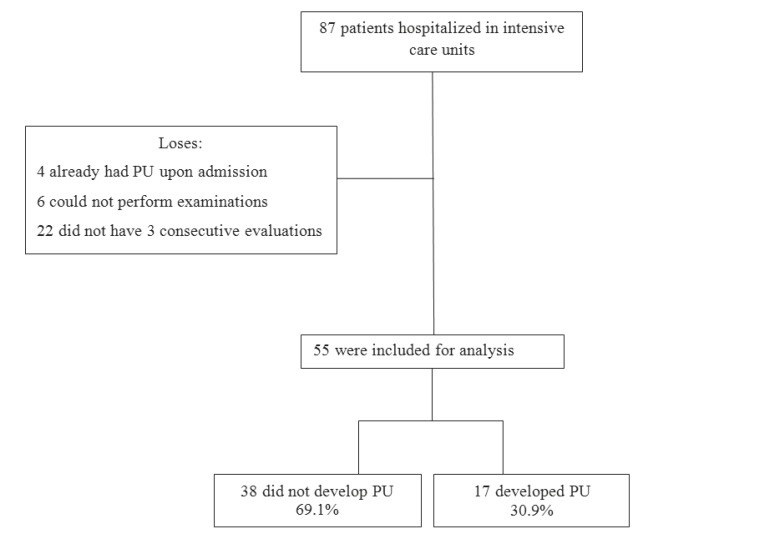
Study participation flowchart. Vitória, ES, Brazil, 2013

A total of 87 patients were admitted to the units during the study period; of these 4 were excluded due to already having ulcers at the time of data collection, 6 for not having laboratory tests performed, and 22 for not having the minimum of 3 consecutive evaluations. Thus, the study included 55 patients, of whom 17 developed pressure ulcers, corresponding to an incidence of 30.9% (CI 95% 18.3 to 43.5) (Figure 1). 

Of the patients included in the study, 28 (51%) were male, 38 (69%) of white skin color, 33 (60%) married, 35 (64%) with elementary education, and 44 (80%) admitted to the Intensive Care Unit. The age ranged from 19 to 85 years, with a mean of 59.4 years. 

Regarding the clinical variables, the length of hospitalization ranged from 5 to 110 days, with a mean of 16.6 days, 30 (54%) patients remained hospitalized for less than 10 days, 38 (69%) were admitted for surgical reasons, 33 (60 %) with clinical diagnoses of gastrointestinal causes, followed by 11 (20%) with cardiorespiratory causes. The majority did not present co-morbidities, such as diabetes mellitus, smoking, and congestive heart failure, 43 (78%), 37 (67%) and 50 (91%), respectively. The majority of the patients also did not use mechanical ventilation, norepinephrine or sedation, 34 (62%), 43 (78%) and 45 (82%), respectively. 

Detection of PUs was found from the 1^st^ to the 19^th^ day of hospitalization, with a mean time of 4.47 days for the appearance. A total of 32 ulcers were identified, 23 (72%) in class I, 15 (47%) in the sacral region, ranging from 1 to 4 ulcers per patient; 9 (53%) of the patients developed at least one ulcer, with a mean of 1.88 PUs per patient.

Regarding the use of the risk assessment scales, it was found that, according to the Waterlow Scale, the patients obtained a mean score of 15.49 points for the total score, ranging from 6 to 26 points. The mean values at the first, second and third evaluations were 16.6, 16.2 and 13.6 points, respectively, with the patients classified as high risk according to this scale.

The mean score obtained for the Braden Scale was 12.8 points for the total score, ranging from 6 to 22 points. The mean scores in the three first evaluations were 12.4, 12.8 and 13.6 points, respectively. Therefore, the majority of the patients were classified as having a moderate risk for developing PUs.

The data in Tables 1 and 2 present the results of the diagnostic tests for the risk assessment scales applied.

**Table 1 -:** Results of diagnostic tests applied to the cutoff scores of the Waterlow scale, according to the evaluation. Vitória, ES, Brazil, 2013

	Score	Sensitivity	Specificity	Likelihood ratio for a negative test (LR-)	Likelihood ratio for a positive test (LR+)
1^st^ evaluation	16	71	47	1.34	0.62
2^nd^ evaluation	15	71	42	1.21	0.70
3^rd^ evaluation	14	88	50	1.76	0.23

**Table 2 -:** Results of diagnostic tests applied to the cutoff scores of the Braden scale, according to the evaluation. Vitória, ES, Brazil, 2013

	Score	Sensitivity	Specificity	Likelihood ratio for a negative test (RV-)	Likelihood ratio for a positive test (LR+)
1^st^ evaluation	12	41	21	0.52	2.79
2^nd^ evaluation	12	53	39	0.87	1.19
3^rd^ evaluation	11	41	18	0.50	3.19

In the first evaluation of the Waterlow scale, the tests detected that the score of 16 presented the best balance between sensitivity (71%) and specificity (47%). In the second evaluation, the score was 15 (sensitivity 71% and specificity 42%), and in the third evaluation, the score was 14 (sensitivity 88% and specificity 50%).

**Figure 2 -:**
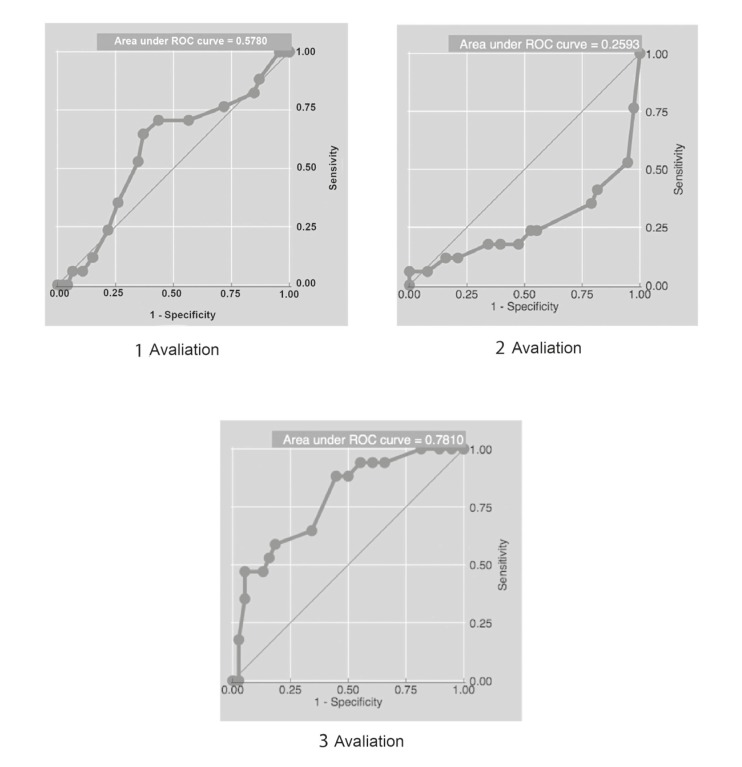
ROC curves for the cutoff scores of the Waterlow scale with critically ill patients, according to the evaluation. Vitória, ES, Brazil, 2013

Considering that the ROC curve is a graphical representation of the true positive values (sensitivity) on the ordinate and the false positive values (specificity) on the abscissa as a function of each cutoff point, the evaluation of the curve of the Waterlow scale showed that it was better for predicting patients at risk for pressure ulcers (Figure 2).

When analyzing the Braden scale, in the first evaluation, the tests detected the score 12 as having the best balance between sensitivity (41%) and specificity (21%). In the second evaluation, the score of 12 remained, with 53% sensitivity and 39% specificity, and in the third evaluation, the score of 11 showed a better balance between sensitivity (41%) and specificity (18%).

**Figure 3 -:**
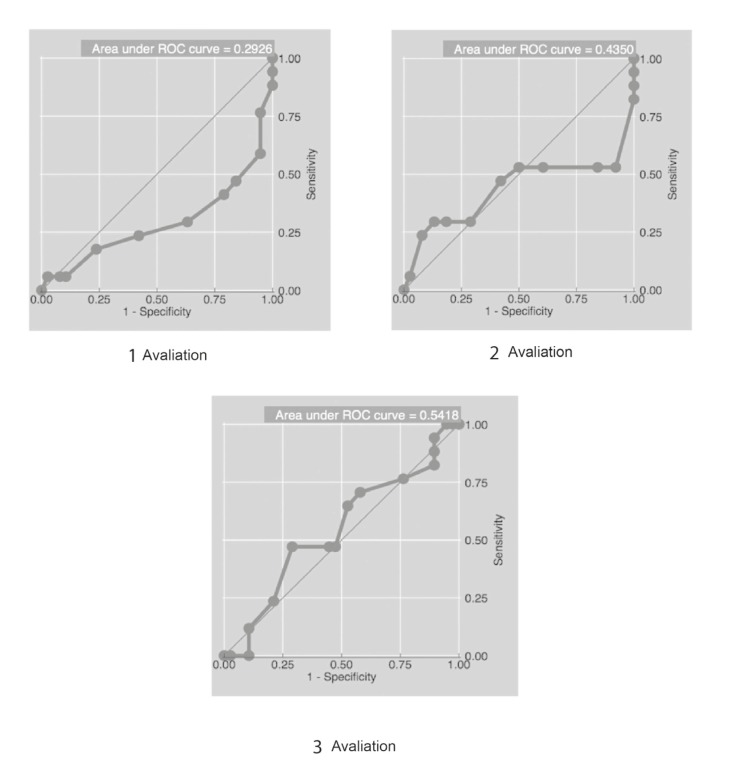
ROC curves for the cutoff scores of the Braden scale with critically ill patients, according to the evaluation. Vitória, ES, Brazil, 2013

For the Braden scale, the evaluation of the ROC curve (Figure 3) showed that it did not present a good prediction of risk of the patient developing pressure ulcers.

## Discussion

The results demonstrate a high incidence of pressure ulcers, in agreement with national publications, which also show a high incidence, especially in critically ill patients. Lower rates are, however, presented in international studies, highlighting the importance of the prevention and monitoring of this injury^(^ 4 ^-^ 5 ^)^. It is believed that the impact of these measures is the reason for the international percentages being much lower than those presented in the national literature^(^ 15 ^)^. 

The results also showed a predominance of surgical patients, with disorders due to gastrointestinal causes, with few days hospitalized in the ICU and with a average time of approximately four days for the appearance of PUs, thus reconfirming the importance of prevention and periodic monitoring of the patient from admission and, even more importantly, the systematic evaluation by the nurse of the risk factors in each patient. The occurrence of PUs in patients in the postoperative period is presented in another study, in which variables related to surgical patients, such as length of anesthesia and extent of the surgery, were predictive for the development of PUs^(^ 16 ^)^.

In relation to the risk assessment, the scales are used to guide the practice, with several existing models, which analyze the items marked to obtain scores that direct the implementation of preventive measures appropriate for the level of individual risk, however, the scales do not include some of the common risk factors for critically ill patient, factors that are not controllable and, therefore, not totally preventable^(^ 17 ^)^. It should be noted that the clinical and metabolic conditions of critically ill patients are often seriously compromised, which enhances the development of PUs. 

Studies that separately analyzed the Braden and Waterlow scales, also with critically ill patients, observed different sensitivities and specificities between them^(^ 10 ^,^ 12 ^,^ 18 ^-^ 19 ^)^. In this study, both scales presented higher sensitivities and lower specificities. The Braden scale presented good sensitivity, however, the specificity was lower, characterizing a good screening instrument; the Waterlow scale presented a better balance between sensitivity and specificity, showing it to be a better instrument for the prediction of risk in this clientele. The cutoff scores were lower than those presented in previous studies for the Braden scale^(12,18-19) ^and similar for the Waterlow scale^(^ 10 ^)^, perceived through factors that this scale evaluates, such as length of surgery, skin type and age.

Studies were found in the literature^(20-21) ^that identified problems in the predictive power of risk assessment scales and affirmed the importance, or rather the relevance of the knowledge and clinical experience of the nurse^(^ 20 ^)^. In clinical practice, these instruments are valid to highlight the vulnerable aspects, to reinforce the need for continuous evaluation and to stimulate prevention, however, these instruments should be tested in the populations in which they will be used and should be applicable to the performance scenario^(^ 5 ^)^. 

The determination of the presence of risk in critically ill patients is extremely challenging for nurses because, in many situations, the factors, such as age, comorbidities, and clinical conditions, among others, are not modifiable. An absence of studies that address the problems related to extrinsic factors can still be observed, considering that the use of quality indicators revealed by the use of scales does not preclude the use of good nursing practices, regarding special care with change of decubitus, with the skin, with the angle of the patient in the bed, how the patient is maneuvered, with the systematic change of the diaper of the patient, especially those that require more than five changes of the diaper within 24 hours, with the use of pads, that is, the use of the therapeutics emphasizing preventive actions^(^ 6 ^)^. Thus, the use of scales, even though they have not been shown to be good risk predictors, can support the professional in the documentation of elements that favor the development of PUs. 

This study presented some limitations: firstly, the temporary closure of the emergency department of the institution during the data collection period led to a small sample size, which may have affected the identification of possible risk factors. Secondly, the use of a single study site does not allow the generalization of the results.Conversely, it should be considered that the data collection being conducted by a trained nurse with expertise in the issue was a strength of the study. The importance of the study for the institution should also be noted, as this university hospital addresses the subject in precursory way, a fact made more relevant given the current moment of change in the care and services management process taking place.

## Conclusion

The study found that the incidence of pressure ulcers in the study population was high. Regarding the performance of both the Braden and Waterlow scales, they presented high sensitivity and low specificity in the three evaluations. The cut off scores found in the first, second and third evaluations were 12, 12 and 11 for the Braden scale, and 16, 15 and 14 for the Waterlow scale.

The scales presented different performance in this sample, with it being found that the Waterlow scale was able to demonstrate better predictive value. Thus, in the clinical practice of the hospital where this study was developed, the use of this scale is suggested as a risk assessment protocol for the identification of patients at risk and the immediate implementation of prevention actions. It is also noteworthy that the Braden scale was shown to be a good screening method; however, this study can be further extended, in order to verify the scale with better prediction and acceptance in the clinical practice among professional nurses of the institution.

Therefore, it is suggested that further, well designed, studies be carried out with these instruments using larger samples and other types of patients, thus contributing to the correct determination of risk for PUs and improved prevention.
